# Transition from BOS to RAS impairs prognosis after lung transplantation—CLAD subtype analysis by CT volumetry

**DOI:** 10.1371/journal.pone.0275563

**Published:** 2022-10-12

**Authors:** Laura Peräkylä, Antti Nykänen, Anneli Piilonen, Risto Kesävuori, Maija Halme, Peter Raivio

**Affiliations:** 1 Department of Cardiac Surgery, Heart and Lung Center, Helsinki University Hospital and University of Helsinki, Helsinki, Finland; 2 Department of Radiology, Medical Imaging Center, Helsinki University Hospital and University of Helsinki, Helsinki, Finland; 3 Department of Pulmonary Medicine, Heart and Lung Center, Helsinki University Hospital and University of Helsinki, Helsinki, Finland; Imperial College Healthcare NHS Trust, UNITED KINGDOM

## Abstract

**Background:**

Chronic lung allograft dysfunction (CLAD), subclassified into bronchiolitis obliterans syndrome (BOS) or restrictive allograft syndrome (RAS), limits survival after lung transplantation. Information concerning transition from BOS to RAS is limited. We aimed to characterize the lung volume change after BOS diagnosis by computed tomography (CT) volumetry and to determine the incidence, risk factors and clinical significance of BOS to RAS transition.

**Methods:**

CT volumetry measurements were performed from 63 patients with CLAD initially classified as BOS by CT volumetry. BOS patients with lung volume remaining >85% of baseline were classified as persistent BOS, whereas BOS patients whose lung volume permanently decreased to ≤85% of baseline were classified as BOS to RAS transition.

**Results:**

During follow-up (median 9.8 years) eight patients (12.7%) were classified as BOS to RAS transition, which decreased recipient (p = 0.004) and graft survival (p = 0.020) in comparison to patients with persistent BOS. Opacities on chest imaging preceded BOS to RAS transition in 88% of patients. Opacities on chest imaging at BOS diagnosis and early CLAD diagnosis after transplantation were risk factors for transition.

**Conclusion:**

Based on lung volume decrease measured by CT volumetry, a small proportion of BOS patients transitioned to RAS which had an adverse effect on recipient and graft survival.

## Introduction

While survival after lung transplantation has improved, the prognosis of patients who develop chronic lung allograft dysfunction (CLAD) remains poor [1,2]. CLAD is an umbrella term for a variety of subtypes, of which bronchiolitis obliterans syndrome (BOS) has obstructive physiology and restrictive allograft syndrome (RAS) has restrictive physiology. The prognosis of patients affected by RAS is significantly worse, with a median survival of 6–18 months after diagnosis, in comparison to a median survival of 3 years of patients diagnosed with BOS [3,4]. A recent International Society for Heart and Lung Transplantation (ISHLT) consensus report recommended that in patients with ≥20% decline in forced expiratory volume in one second (FEV1), RAS should be defined by a concomitant ≥10% decline in total lung capacity (TLC) measured by body plethysmography and persistent opacities on chest imaging [5]. TLC measurement, however, is not routine in all lung transplantation centers, and not all patients are capable of undergoing body plethysmography measurement. Forced vital capacity (FVC) measurements have been used as a surrogate for TLC to measure restriction, but this technique is susceptible for being affected by air trapping.

As first reported by Saito et al., computed tomography (CT) volumetry can be employed as an alternative method for subtyping CLAD [6]. We also recently demonstrated that lung volume decrease measured by CT volumetry identified patients at risk for graft loss after lung transplantation [7]. However, only limited information is available on the progression of lung volumes after the initial CLAD diagnosis. Moreover, data concerning possible transition from the BOS phenotype to RAS phenotype, and its clinical significance, is scarce. In the present study, we aimed to characterize the pattern of lung volume change after BOS diagnosis by CT volumetry and to determine the incidence, risk factors and clinical significance of BOS to RAS transition.

## Materials and methods

### Study design and population

**Fig 1:**
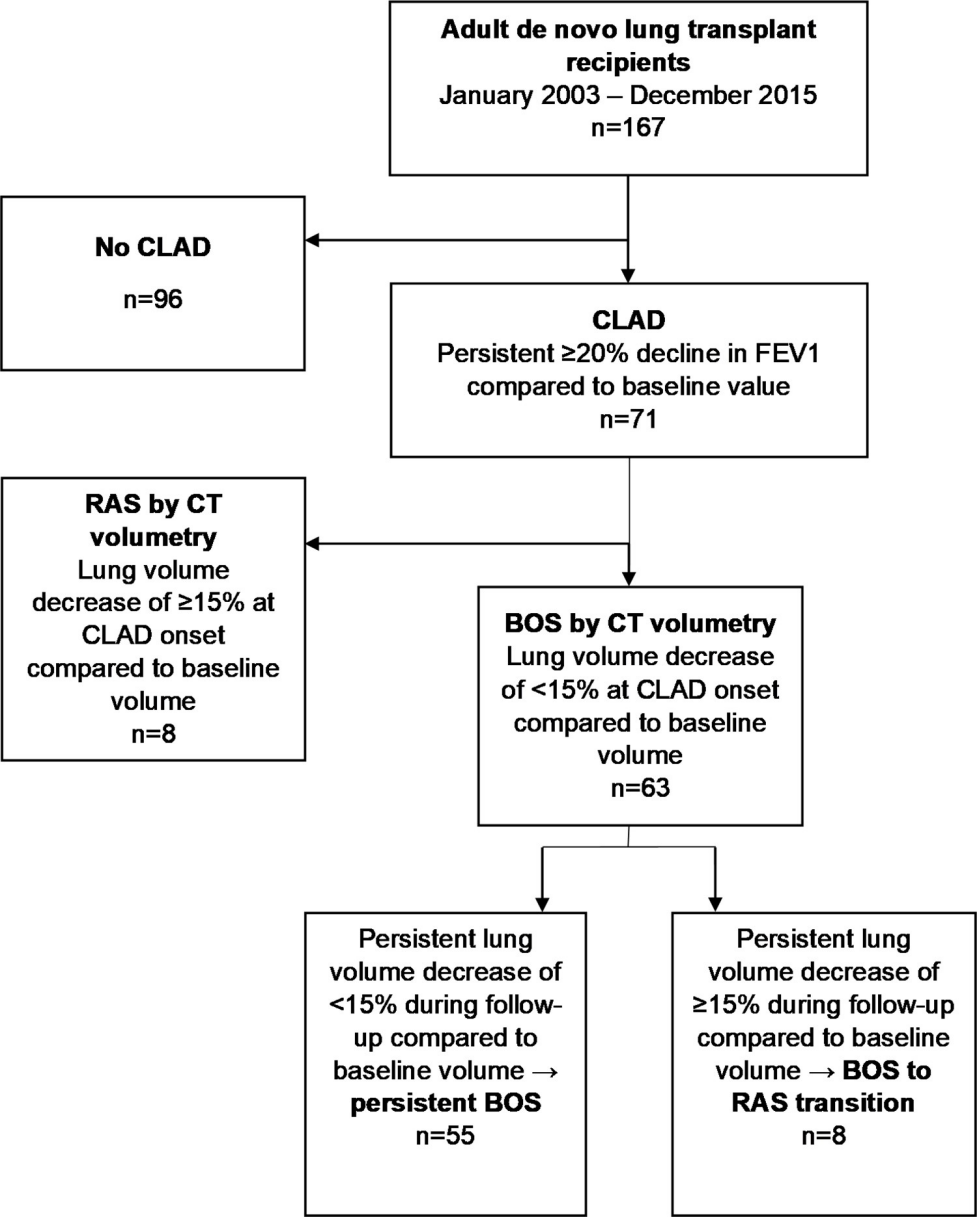
Flow chart of patient classification during study. BOS: Bronchiolitis obliterans syndrome; CLAD: Chronic lung allograft dysfunction; FEV1: Forced expiratory volume in one second; FVC: Forced vital capacity; RAS: Restrictive allograft syndrome.

The retrospective study cohort consisted of 167 adult de novo lung transplant recipients transplanted in the Helsinki University Hospital, Helsinki, Finland between January 2003 and December 2015. We have previously reported on the clinical results of this patient cohort and on the CT volumetry findings at CLAD onset [7,8]. During follow-up, 71 (40.6%) patients from the cohort were diagnosed with CLAD based on a persistent decline of FEV1 in comparison to baseline [8]. The current patient follow-up lasted until January 2021. Median follow-up time per patient after lung transplantation was 9.8 years. Patients were previously subcategorized into BOS and RAS based on lung volume change in the CLAD onset CT in comparison to baseline volume [7]. The study population of the present study consisted of the 63 (88.7%) patients who had a lung volume decrease of <15% compared to baseline at CLAD onset CT and were thus classified as having BOS (Fig 1). The patients underwent serial CT scans as part of their follow-up after lung transplantation, with routine CT imaging performed at 1, 2, 3, 4, 6, 9, 12, 18 and 24 months after transplantation, and yearly thereafter. Additional CT studies were performed if clinically indicated.

Patient data was collected from electronic patient records. The study was reviewed and approved by the Institutional Review Board of the Helsinki University Hospital, Helsinki, Finland. As the study was a retrospective study, The Institutional Review Board concluded that informed consent from the patients would not be required. The study was performed in accordance with the 2000 Declaration of Helsinki and the Declaration of Istanbul 2008.

### CLAD diagnosis and subtyping

CLAD was diagnosed by a persistent ≥20% decline in FEV1 from baseline, defined as the mean of two best postoperative FEV1 measurements, when other causes of FEV1 decline were absent. CLAD subtyping was conducted based on lung volume as suggested by Saito et al. [6]. The CT closest to the date of CLAD diagnosis was chosen as the CLAD onset CT. The CT closest to the date of the best FEV1 value was chosen as the baseline CT. Lung volume was measured from CTs obtained at maximal inspiration using a dedicated image processing software (Vitrea, Vital Images Inc., Minnetonka, MI, USA). Semi-automatic three-dimensional segmentation of both lungs was performed to calculate lung volume. The software also provides an analysis of mean lung density. The contours of the lungs were traced manually if their automatic recognition by the software was inaccurate. A lung volume decrease of <15% in the CLAD onset CT compared to baseline CT volume led to classification as BOS. A lung volume decrease of ≥15% led to classification as RAS. Presence of opacities on CT images was evaluated by an experienced thoracic radiologist.

Patients subtyped as BOS at CLAD onset were followed for a median of 5.1 years until January 2021. During follow-up, CT volumetry analysis was conducted to further classify the patients into BOS to RAS or persistent BOS according to volume changes. CT imaging was conducted according to a standardized lung transplantation patient protocol. A median of 5 CT images were analyzed per patient.

As we have reported in an earlier study, at CLAD onset 63 (88.7%) patients were classified as BOS (lung volume decrease of <15% compared to baseline) and 8 (11.3%) patients were classified as RAS (lung volume decrease of ≥15% compared to baseline) (Fig 1). Patients were reclassified as BOS to RAS transition if lung volume in CT volumetry compared to baseline decreased permanently by ≥15% during follow-up. Other reasons for volume change were also considered during the analysis and this led to the exclusion of 8 patients from the BOS to RAS group. In 4 patients, the reason for volume decline was hypoventilation; in 3 patients, reappearance of primary disease affecting lung volume and, in 1 patient, pancreatitis affecting lung expansion.

### Radiological assessment

CT scans were performed with a multidetector CT scanner using a standardized lung transplant imaging protocol. Following the technical development of the scanners, the protocol and the parameters varied during the follow-up period. Initially, the protocol included a helical CT scan, high-resolution CT (HRCT) scans and expiratory HRCT scans at spaced intervals. Later, standard helical slices and volumetric HRCT scans were reconstructed from the same inspiratory series. Expiratory HRCT scans were obtained at the beginning at three levels, later at 20 mm intervals. Lungs were imaged at full inspiration and expiration without intravenous contrast agent. The CT scans were analyzed for presence of opacities and air trapping for this study by an experienced thoracic radiologist.

### Classification of CLAD subtypes according to the 2019 consensus report from the Pulmonary Council of the ISHLT

The CLAD subtype classification based on CT volumetry was compared to the classification recommended by the recent 2019 consensus report of the Pulmonary Council of the ISHLT [9]. The patients were classified as BOS, RAS, mixed, undefined, and unclassified subtypes as outlined by the 2019 consensus report. Obstruction was defined as FEV1/ FVC ratio <0.70. Restriction was defined as FVC≤80% of baseline as previously described by Levy et al. [10]. Baseline FVC for restriction was defined as the mean of the FVC values taken at the time of best postoperative FEV1 values. Opacities on chest imaging were defined, according to the consensus report, as “persistent ground glass, consolidation, small linear and reticular opacities on CT imaging, multilobar and/or showing increasing pleural thickening consistent with a diagnosis of pulmonary and/or pleural fibrosis” [9].

### Statistical analysis

IBM SPSS Statistics 27 software was employed for statistical analysis. For comparisons between groups, Fisher’s exact test was used for categorical variables. The Student’s t-test was used for parametric continuous variables. The Mann-Whitney U test was used for non-parametric variables. Differences in survival were analyzed by using Kaplan-Meier log rank test. Further, Cox proportional hazards regression analysis was performed to identify risk factors for BOS to RAS transition. Recipient and donor characteristics, radiological, spirometric and lung volumetric covariates present at CLAD onset were analyzed with a univariate analysis. Covariates from the univariate analysis with a p≤0.20 were included in the multivariate analysis by way of block entry. P<0.05 was considered significant.

## Results

### A small proportion of BOS patients transitioned to RAS after CLAD onset

**Fig 2:**
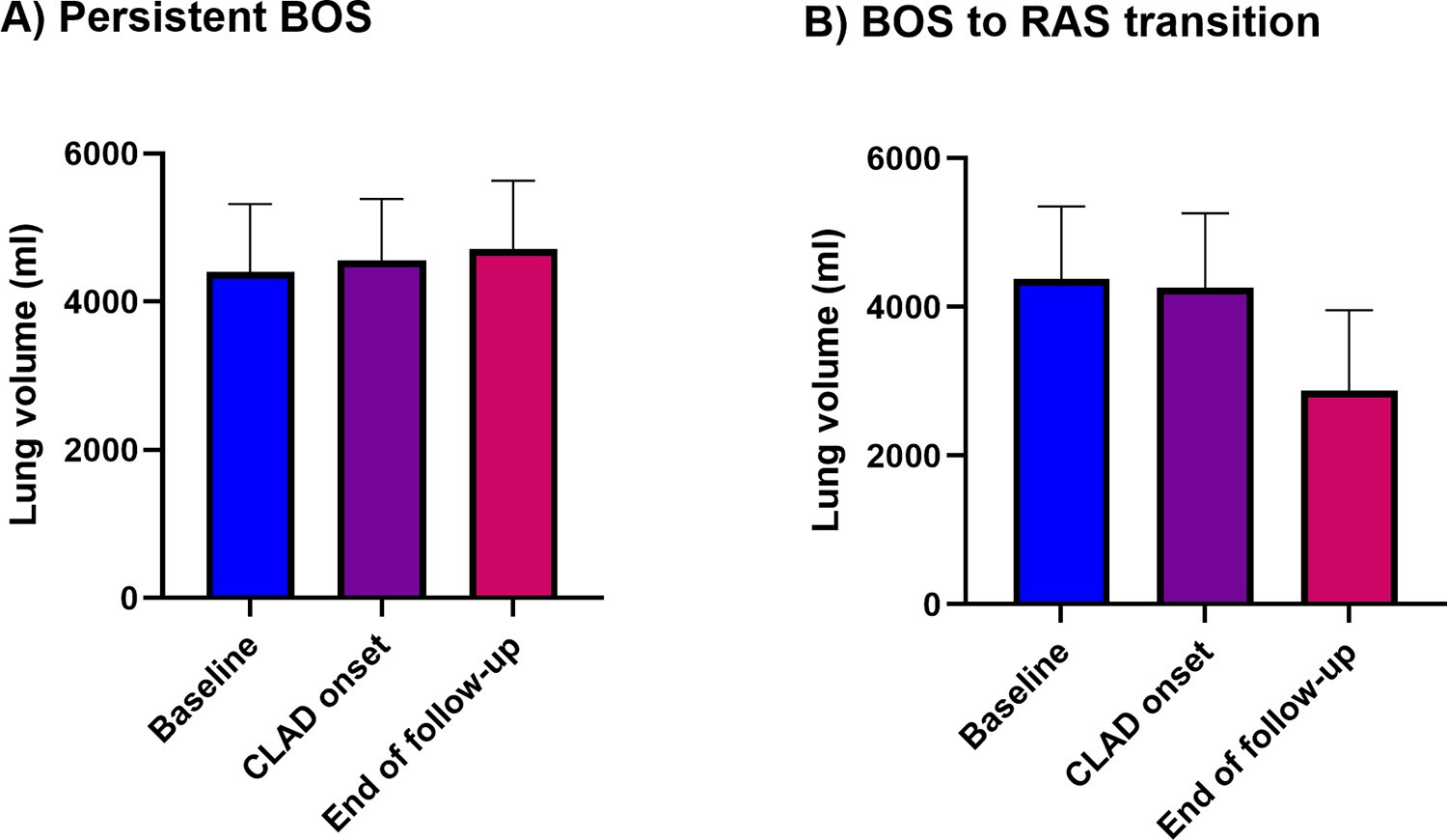
Lung volume progression during follow-up. A) Lung volume during follow-up for persistent BOS, B) lung volume during follow-up for BOS-to-RAS transition. Data presented as median with interquartile range. BOS: Bronchiolitis obliterans syndrome; CLAD: Chronic lung allograft dysfunction; RAS: Restrictive allograft syndrome.

During follow-up after CLAD onset, 8 (12.7%) of the 63 patients initially diagnosed as BOS were reclassified as RAS as their lung volume permanently decreased ≥15% compared to baseline volume (BOS to RAS transition). There was no significant difference in lung volumes between persistent BOS and BOS to RAS transition groups at CLAD onset (median lung volume in BOS 4552 IQR 3487–5387 ml, and in BOS to RAS 4262 IQR 3877–5261 ml, p = 0.99) (Fig 2). During follow-up lung volumes of patients in the persistent BOS group increased, however the change in lung volume was not statistically significant (median 4552 ml at CLAD onset vs. median 4715 ml at latest follow-up), while lung volumes of patients in the BOS to RAS transition group decreased by 32.5% (median 4262 ml at CLAD onset vs. median 2877 ml at latest follow-up) (Fig 2).

The transplantations of the patients with BOS to RAS transition were dispersed throughout the study period, and were performed between 2006 and 2015. BOS to RAS transition occurred at a median of 26.7 IQR 14.4–49.7 months after CLAD diagnosis. At CLAD diagnosis, patients in the BOS to RAS transition group had a significantly lower FEV1/FVC ratio compared to patients in the persistent BOS group (median 0.56 IQR 0.50–0.66 liters versus 0.64 IQR 0.56–0.73 liters, p = 0.048). There was no significant difference in baseline FVC between the persistent BOS and BOS to RAS transition groups (median 3.54 (IQR 2.60–4.48) and 3.41 (IQR 2.57–4.31) liters, respectively, p = 0.86). At CLAD onset, 14/55 (25%) of patients with persistent BOS and 2/8 (25%) patients with BOS to RAS transition had an FVC decline >20% from baseline FVC. The median FVC at CLAD onset as a proportion of baseline FVC was 87% for the persistent BOS group and 89% for the BOS to RAS transition group (p = 0.84). At the end of follow-up, 34/55 (62%) of persistent BOS and 6/8 (75%) of BOS to RAS transition patients had an FVC decline >20% from baseline FVC.

There was no significant difference in the proportion of extended criteria donors between the groups (persistent BOS vs BOS to RAS transition p = 0.47), and also other recipient or donor characteristics were similar (Tables 1 and 2).

**Table 1:** Recipient and donor characteristics, time to CLAD onset and BOS to RAS transition.

						CLAD status	
					Persistent BOS	BOS to RAS transition	
Variables					n = 55	n = 8	p-value
Recipient characteristics							
	Recipient age in years, median (IQR)				56 (46–71)	56 (50.8–61.5)	0.66
	Sex male, n (%)				34 (61.82)	4 (50.00)	0.70
	Primary diagnosis						
		Emphysema/COPD, n (%)			10 (18.18)	3 (37.50)	0.35
		Cystic fibrosis, n (%)			0	0	NA
		Idiopathic pulmonary fibrosis, n (%)			28 (50.91)	3 (37.50)	0.71
		Alpha-1 antitrypsin deficiency, n (%)			6 (10.91)	0	1.00
		Primary pulmonary hypertension, n (%)			3 (5.45)	0	1.00
		Other, n (%)			8 (14.55)	2 (25.00)	0.60
	Recipient history of smoking, n (%)				34 (61.82)	5 (62.50)	1.00
	Any biopsy-proven rejection after transplantation, n (%)				32 (58.18)	4 (50.00)	0.72
	Greater or equal to 4 HLA mismatches in HLA-A, B or DR, n (%)				38 (69.09)	8 (100.00)	0.10
	Tacrolimus as primary immunosuppression, n (%)				4 (7.27)	0	1.00
	Cyclosporine A as primary immunosuppression, n (%)				51 (92.73)	8 (100.00)	1.00
Donor characteristics							
	Donor age in years, median (IQR)				46.0 (39–56)	46.5 (42.5–52.3)	0.99
	Sex male, n (%)				24 (43.64)	4 (50.00)	1.00
	Standard vs extended criteria donor						0.47
		Standard, n (%)			26 (47.27)	5 (62.50)	
		Extended, n (%)			29 (52.73)	3 (37.50)	
	CMV D+/R-, n (%)				7 (12.73)	1 (12.50)	1.00
Time (months) from transplantation to CLAD diagnosis, median (IQR)					31.4 (17.5–56.7)	16.9 (12.8–24.3)	0.06
Time (months) from CLAD diagnosis to BOS to RAS transition, median (IQR)						26.7 (14.4–49.7)	
Time (days) between baseline by spirometry and baseline CT, median (IQR)					0 (0–3)	0 (0–0)	
Time (days) between CLAD diagnosis and CLAD CT, median (IQR)					0 (-1-0)	0 (-0.75–0)	

Data analyzed by Fisher’s exact test for categorical variables. Student’s t-test used for parametric continuous variables. Mann-Whitney U -test used for nonparametric continuous variables.

**Table 2:** Pulmonary function tests at CLAD onset.

					CLAD status		
				Persistent BOS		BOS to RAS transition	
Variables				n = 55		n = 8	p-value
FEV1/FVC at CLAD diagnosis, median (IQR)				0.64 (0.56–0.73)		0.56 (0.50–0.66)	0.048
FEV1 at CLAD diagnosis, median (IQR)				1.70 (1.41–2.32)		1.47 (1.23–1.94)	0.296
FVC at CLAD diagnosis, median (IQR)				2.79 (2.28–3.86)		2.64 (2.38–3.75)	0.84

Student’s t-test used for parametric continuous variables. Mann-Whitney U -test used for nonparametric continuous variables.

### Opacities on chest imaging preceded BOS to RAS transition

At CLAD onset, 3 out of 8 (38%) patients in the BOS to RAS transition group presented opacities on lung CT scan while opacities on chest imaging were present in only one out of 55 (2%) patients in the persistent BOS group (p = 0.005) (Table 3). During follow-up, opacities on chest imaging were detected in 10 (18%) patients in the persistent BOS group. Altogether, opacities were present in 7 (88%) patients prior to BOS to RAS transition. In 5 (71%) patients with BOS to RAS transition, opacities on chest imaging were located apically and in 2 (29%) patients diffusely. In 4 out of 7 (57%) patients the opacities were radiologically interpreted as pleuroparenchymal fibroelastosis. Air trapping was common in both BOS to RAS transition (88%) and persistent BOS (98%) during follow-up.

**Table 3:** Radiological analysis.

									CLAD status		
								Persistent BOS		BOS to RAS transition	
Variables								n = 55		n = 8	p-value
Opacities on chest imaging											
	Presence of opacities at CLAD onset, n (%)							1 (1.82)		3 (37.50)	0.005
	Presence of opacities at any stage after CLAD onset, n (%)							10 (18.20)		7 (87.50)	<0.001
	Presence of opacities prior to BOS to RAS transition, n (%)									7 (87.50)	
	Localisation of opacities in BOS to RAS transition										
		Apical, n (%)								5 (71.40)	
		Diffuse, n (%)								2 (28.60)	
	Radiological interpretation of PPFE in BOS to RAS transition, n (%)									4 (57.10)	
Air trapping											
	Presence of air trapping at CLAD onset, n (%)							32 (58.18)		4 (50.00)	0.05
	Presence of air trapping at any stage after CLAD onset, n (%)							54 (98.18)		7 (87.50)	0.24
	Presence of air trapping prior BOS to RAS transition, n (%)									7 (87.50)	
Mean lung density at CLAD onset, HU (SD)								-807.5 (42.3)		-812.5 (20.7)	0.75
Mean lung density at end of follow-up, HU (SD)								-829.3 (44.3)		-746.8 (56.9)	<0.001

Categorical variables analyzed with Fisher’s exact test. Parametric continuous variables analyzed with Student’s t-test. Nonparametric continuous variables analyzed with the Mann-Whitney U -test.

There was no difference in mean lung density between persistent BOS and BOS to RAS transition at CLAD onset (Table 3). At the end of follow-up, mean lung density in the BOS to RAS transition group was significantly higher than in persistent BOS group (-746.8 HU vs -829.3 HU, respectively, p≤0.001).

### Transition from BOS to RAS had a detrimental effect on survival

**Fig 3:**
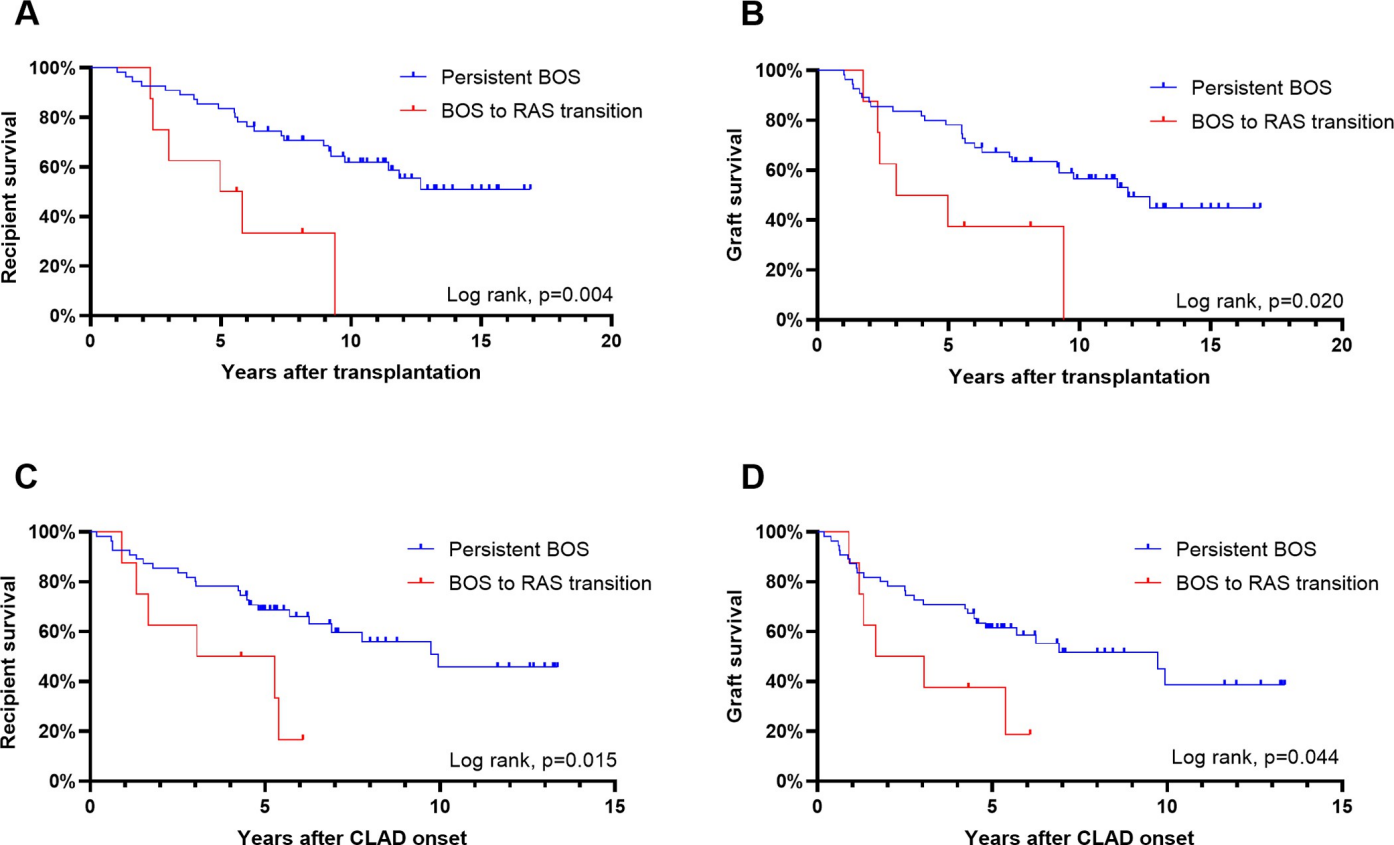
Impact of class change on survival. A) Recipient survival after lung transplantation, B) graft survival after lung transplantation, C) recipient survival after CLAD onset and D) graft survival after CLAD onset. Impact analyzed with Kaplan-Meier survival curves using the log rank -test. BOS: Bronchiolitis obliterans syndrome; CLAD: Chronic lung allograft dysfunction; RAS: Restrictive allograft syndrome.

BOS to RAS transition patients had significantly worse patient (p = 0.004, Fig 3A) and graft survival after transplantation (p = 0.015, Fig 3B) than recipients in the persistent BOS group. The same was true when comparing patient (p = 0.02, Fig 3C) and graft survival (p = 0.044, Fig 3D) after CLAD onset. After CLAD onset, the median survival estimate for patients in the BOS to RAS transition group was limited to 3.0 years in comparison to 9.9 years in the persistent BOS group.

### Risk factors for BOS to RAS transition

In univariate Cox regression analysis, early CLAD diagnosis after transplantation, lower FEV1/FVC ratio at CLAD onset and the presence of opacities on chest imaging at CLAD onset presented as risk factors for BOS to RAS transition (Table 4A).

**Table 4:** a. Cox regression analysis on risk factors for BOS to RAS transition. b. Multivariate Cox regression analysis on risk factors for BOS to RAS transition.

Variables		Hazard ratio (HR)	95 CI for HR	p-value
**UNIVARIATE ANALYSIS**				
Recipient characteristics				
	Recipient age	1.01	0.94–1.10	0.74
	Recipient sex, male	0.92	0.23–3.70	0.91
	Recipient history of smoking	1.01	0.24–4.25	0.99
	Primary diagnosis emphysema/COPD	1.64	0.39–6.93	0.50
	Primary diagnosis cystic fibrosis	NA	NA	NA
	Primary diagnosis idiopathic pulmonary fibrosis	0.91	0.21–3.86	0.89
	Primary diagnosis alpha-1 antitrypsin deficiency	0.04	0.00–503.96	0.50
	Primary diagnosis pulmonary hypertension	0.05	0.00–21622.02	0.64
	Any biopsy-proven rejection after transplantation	0.90	0.22–3.67	0.88
	Greater or equal to 4 HLA mismatches in HLA-A, B or DR	25.97	0.01–122924.90	0.45
	Cyclosporine A as primary immunosuppression	21.01	0.00–7.820 E+12	0.82
	Tacrolimus as primary immunosuppression	0.48	0.00–1.772E+10	0.82
	Time (in months) from transplantation to CLAD diagnosis	0.94	0.89–1.00	0.04
Donor characteristics				
	Donor age	1.00	0.94–1.06	0.97
	Donor sex, male	1.31	0.32–5.32	0.71
	Donor history of hypertension	0.04	0.00–186.62	0.45
	CMV D+/R-	1.31	0.16–10.68	0.80
	Extended criteria donor	0.60	0.14–2.54	0.48
Spirometry at CLAD onset				
	FEV1 at CLAD diagnosis, L	0.52	0.16–1.66	0.27
	FVC at CLAD diagnosis, L	0.89	0.40–2.02	0.79
	FEV1/FVC at CLAD diagnosis	0.003	0.00–2.03	0.08
Radiological findings at CLAD onset				
	Presence of air trapping at CLAD onset	0.82	0.20–3.32	0.78
	Presence of opacities at CLAD onset	6.30	1.50–26.48	0.01
Volumetry at CLAD onset				
	Lung volume at CLAD onset, mL	1.00	0.99–1.01	0.61
	Lung volume change from baseline, mL	1.00	0.99–1.01	0.45
Mean lung density at CLAD onset, HU		1.00	0.98–1.03	0.68
**Variables**		Hazard ratio (HR)	95 CI for HR	p-value
**MULTIVARIATE ANALYSIS**				
	Time (in months) from transplantation to CLAD diagnosis	0.92	0.84–0.99	0.03
	FEV1/FVC at CLAD diagnosis	0.16	0.00–177.75	0.61
	Presence of opacities on chest imaging at CLAD onset	12.76	1.66–98.13	0.01

Risk factor analysis conducted using Cox proportional hazard regression model. All variables in the univariate analysis (Table 4A) with a p-value ≤ 0.20 were included in the multivariate analysis (Table 4B).

In multivariate analysis, early CLAD diagnosis after transplantation (time from transplantation to CLAD onset, HR 0.92, p = 0.03), and presence of opacities on chest imaging at CLAD onset (HR 12.8, p = 0.01) were risk factors for BOS to RAS transition (Table 4B).

### Classification of CLAD subtypes according to the 2019 consensus report from the Pulmonary Council of the ISHLT

The 63 CLAD patients were classified according to the classification guidelines recommended in the consensus report by the ISHLT (S1 File). (9) At CLAD diagnosis, 31 (49%) patients were classified as BOS, 17 (27%) as undefined and 15 (24%) unclassified. Applying the same classification guidelines at the end of follow-up, 16 (25%) patients were classified as BOS, 13 (21%) patients as mixed and 27 (43%) patients as undefined. Seven (11%) patients remained unclassified at the end of follow-up.

## Discussion

We studied the progression of lung volumes in lung transplant recipients diagnosed with BOS employing CT volumetry. The main findings of the present study were: 1) During the follow-up, lung volumes of over 10% of patients initially diagnosed with BOS decreased to warrant phenotype reclassification. 2) BOS to RAS transition significantly impaired patient and graft survival. 3) Presence of opacities on chest imaging at CLAD onset and early CLAD diagnosis after lung transplantation were risk factors for BOS to RAS transition.

Strikingly, BOS to RAS transition defined by CT volumetry had a major detrimental impact on survival in our cohort. Eight (12.7%) patients initially classified as BOS were reclassified as RAS during the follow-up, and 6 (75%) of these BOS to RAS transition patients died during the follow-up. Our findings thus closely resemble the findings by Van Herck et al, [11] who defined CLAD subtypes using TLC and FVC. Similarly to our findings, Van Herck et al. found that 10% of BOS patients evolved from an obstructive to restrictive phenotype, and the median survival of these patients was only 3.2 years after BOS diagnosis. In our study, 62% of patients in the persistent BOS group had an FVC decline ≥20% from baseline, which in part depicts the development of hyperinflation and air trapping. The same group also compared patients transitioning from BOS to a mixed phenotype to patients initially classified as RAS, and reported a longer survival in the BOS-to-mixed group. The median survival of patients in the BOS-to-mixed group was 4.1 years after CLAD onset [12]. Collectively, the present and previous results [11,12] indicate that the initial CLAD phenotype may evolve over time in a substantial proportion of patients, and transition from an obstructive to a more restrictive phenotype has a negative clinical impact.

Development of upper-lobe dominant fibrotic changes in the allograft, leading to lung restriction, is a central part of RAS pathogenesis [4,6]. In our study, a third of the patients in the BOS to RAS transition group presented with opacities typically seen in RAS patients (persistent ground glass, consolidation, or interstitial reticular or linear opacities consistent with pulmonary or pleural fibrosis) at CLAD onset CT and 83% had such opacities on chest imaging prior to BOS to RAS transition. According to the 2019 ISHLT consensus statement, patients with opacities at CLAD onset would be classified as undefined subtype. Based on the presence of opacities on chest imaging in patients with RAS, and also in patients that transition from BOS to RAS, fibroproliferative processes may be central in the pathogenesis of both of these CLAD phenotypes. From a clinical point of view, the finding of opacities typically seen in RAS-patients in a patient classified as BOS may be important, prompting clinicians to careful follow-up, which is also highlighted today by classification as the undefined subtype.

Interestingly, early CLAD diagnosis was a risk factor for BOS to RAS transition. Acute rejections have been associated with the development of both BOS and RAS [13]. 50% of the BOS to RAS transition patients in our cohort had had a biopsy-proven rejection after transplantation, but the proportion of patients with acute rejection was, in fact, lower than that of patients in the persistent BOS group. Patients undergoing BOS to RAS transition also had a more obstructive FEV1/FVC ratio at CLAD onset compared to patients in the persistent BOS group. There was, however, no significant difference in FEV1 at CLAD onset between the two groups. In agreement with our finding, Van Herck et al. also reported no significant difference in FEV1 at CLAD diagnosis between BOS and BOS to RAS transition. Following these results, for clinicians, valuable indications for potential BOS to RAS transition are early CLAD onset and presence of opacities on chest imaging at CLAD onset rather than spirometry findings. It remains unclear why earlier CLAD onset increases risk for BOS to RAS transition, and what makes the patients in BOS to RAS potentially susceptible to phenotype change to RAS.

Many other promising CLAD risk factors have been reported for not only CLAD onset but also for phenotype transition. Detected donor specific antibodies have been linked with increased risk of CLAD [14,15]. Increased number of eosinophils in bronchoalveolar lavage fluid samples have been linked to RAS development, whereas early neutrophilia has been linked to BOS onset [14]. Fuchs et al. interestingly reported that in patients transitioning from a non-RAS-like opacity phenotype to a RAS-like opacity phenotype, 47% patients had A1 or higher grade acute cellular rejection between CLAD onset and phenotype transition, which was significantly higher than in any other transition type [16]. In the present study, the proportion of patients with a biopsy-proven rejection in the BOS to RAS transition group was, in fact, lower than in the persistent BOS group. Further, our risk factor analysis of BOS to RAS transition did not find biopsy-proven rejection to be a risk factor for transition. Still, expanding knowledge of potential risk factors for transition might help to detect patients at risk who may potentially be candidates for targeted therapeutic measures.

CT volumetry analysis was initially introduced as an alternative method to subtype CLAD patients by Saito et al. in a retrospective study reporting the accuracy of CT volumetry in distinguishing BOS and RAS [6]. Saito et al. compared different CT volumetry thresholds in patients with 10% TLC decrease and found that a lung volume threshold of <85% was the most accurate threshold (diagnostic accuracy 0.937). CT volumetry has also previously been used in a non-transplantation setting, where a very strong correlation between TLC by plethysmography and CT volumetry was reported [17]. Quantitative CT scan applications and possibilities in CLAD diagnostics are developing. Recently, changes in quantitative density metrics derived from CT scans were associated with discovery of early CLAD [18]. We found that patients with BOS to RAS transition had a significantly higher mean lung density at the end of follow-up, while there was no difference in mean lung density between the two groups at CLAD onset. The higher lung density in the BOS to RAS transition group is likely to represent the development of opacities typically associated with RAS in the allograft. We believe that quantitative CT scan analyses might provide a tool for identifying patients at risk for CLAD at different stages of the post-lung transplant period. The importance of possibilities provided by quantitative CT scan analyses are further highlighted in centers where TLC measurements are not standard protocol.

The mixed phenotype of CLAD was described in the 2019 consensus statement by the ISHLT [9]. It is defined by obstruction by spirometry, a concomitant restriction measured by a decline in TLC and findings of persistent opacities in chest imaging. According to the consensus statement, all cases of CLAD which transition from BOS to RAS will meet the criteria of the mixed phenotype. Restriction in the definition is, however, derived from a TLC decline. In the absence of TLC measurements, FVC may also be used as a surrogate marker for defining restriction. While our study lacks TLC measurements, the restriction is validated by the decline in lung volume measured by CT volumetry. The median FEV1/FVC ratio at CLAD diagnosis of patients with BOS to RAS transition was clearly obstructive. It can therefore be argued that the patients in the BOS to RAS transition group presented with the mixed phenotype of CLAD.

As interestingly reported by Verleden et al, patients with the mixed phenotype exhibited opacities more strongly concentrated to the apical regions (75%) compared to RAS patients (32.5%) [12]. The group further reported that in a pathological assessment of a biopsy, the mixed phenotype patients more frequently had pleuroparenchymal fibroelastosis (70%) as pathology finding compared to RAS (38%), but this finding did not reach statistical significance. In the 2019 Consensus report by the ISHLT, no distinction is made between the locations of opacities, perhaps highlighting the diverse presentation of opacities. Analysing the location of the opacities in our study, we found that five (71%) of BOS to RAS transition patients with opacities on chest imaging had opacities located in the apical regions, whereas in two (29%) patients the opacities had a diffuse presentation. Further radiologically analysing the proportion of pleuroparenchymal fibroelastosis in the opacities apparent on chest imaging, we found that four (57%) out of the seven patients developing opacities in BOS to RAS transition presented with pleuroparenchymal fibroelastosis on lung CT imaging (Table 3) [19]. Our results thus reflect those of Verleden et al., with a predominantly apical presentation of opacities in patients with a combination of the obstructive and restrictive phenotype of CLAD.

We also subclassified the 63 lung transplant patients according to the 2019 consensus report by the ISHLT (S1 File). We have previously used the same classification to assess the clinical applicability of CT volumetric classification of CLAD [7]. Unfortunately, as TLC measurements were not a part of our routine follow-up protocol at the time of the study period, we were unable to use TLC to measure restriction. We defined restriction as FVC decline ≤80% from baseline. At the beginning of follow-up, 49% of patients were classified as BOS, 27% as undefined and 24% as unclassified. At the end of follow-up, the proportion of patients with BOS according to the consensus report declined to 25%, and 21% of patients were classified as mixed. Notably, the proportion of patients classified as undefined increased to 43%. The increase in patients in the undefined group may in part be explained by an FVC decline due to hyperinflation and air trapping [9] portraying the BOS phenotype. The 21% of patients in mixed classification reflects our finding of phenotype transition from BOS to RAS. Yet, the fragmentation of the classification into four subgroups at the end of follow-up depicts the difficulty in the application of the 2019 consensus report classification in a clinical setting [10]. Indeed, the clarity of the CT volumetry-based classification may be a significant advantage but further comparative prospective studies are needed.

There are limitations that apply to this study. Firstly, our retrospective patient cohort from a single-center nationwide unit was relatively small. As such, also the number of patients with BOS to RAS transition remained low. A higher number of patients would improve the risk factor analysis. Moreover, as a nationwide center, the patient base in our cohort was rather homogeneous. For example, the patients diagnosed with BOS at CLAD onset did not include any patients with cystic fibrosis as a primary diagnosis, whereas it makes up a significant proportion of primary diagnoses in many other lung transplant cohorts. [6,11] A multicenter approach could offer valuable additional information. Secondly, due to the long-term nature of the retrospective study, we employed CT scans obtained by multiple CT scanners throughout the study. While there was a consistent center-wide lung transplantation patient protocol in place for imaging throughout the study period, due to technological development of the CT scanners, it is probable that the accuracy of CT scans in the beginning of the study period are substandard compared to the CT imaging technology in place today. Thirdly, we were not able to use TLC to distinguish CLAD subtypes. TLC measurements have not been part of the standardized lung transplant patient study protocol in our institution prior to the ISHLT consensus report published in 2019. TLC measurements would have offered valuable information and validated our findings and enabled us to confirm mixed phenotype diagnoses. Lastly, the retrospective study covered lung transplantation over the course of 12 years, during which clinical treatment of lung transplantation patients can have changed.

In conclusion, our retrospective study characterized the pattern of lung volume change by CT volumetry after CLAD onset and demonstrated the negative impact of BOS to RAS transition on patient and graft survival. During follow-up of BOS patients, the detection of opacities typically seen in RAS-patients on CT imaging may precede transition to RAS and declining lung volume by CT volumetry may suggest a poor prognosis.

## Supporting information

S1 File Cohort classification according to the 2019 Consensus report by the Pulmonary council of the ISHLT. A) Subclassification of CLAD at CLAD onset according to the 2019 Consensus report by ISHLT, B) subclassification distribution at CLAD onset, C) subclassification of CLAD at the end of follow-up according to the 2019 Consensus report by ISHLT, D) subclassification distribution at the end of follow-up.
(DOCX)

S1 Dataset (XLSX)

## References

[1] Verleden S, Ruttens D, Vendermeulen E, Vaneylen A, Dupont L, Van Raemdonck D, et al. Bronchiolitis Obliterans Syndrome and Restrictive Allograft Syndrome: Do Risk Factors Differ? Transplantation. 2013;95:1167–1172. doi: 10.1097/TP.0b013e318286e076 23425818

[2] Yusen RD, Edwards LB, Kucheryavaya AY, Benden C, Dipchand AI, Dobbels F, et al. The Registry of the International Society for Heart and Lung Transplantation: Thirty-first Adult Lung and Heart-Lung Transplant Report– 2014; Focus Theme: Retransplantation. J Heart Lung Transplant. 2014;33:1009–1024. doi: 10.1016/j.healun.2014.08.004 25242125

[3] Verleden SE, Ruttens D, Vandermeulen E, Bellon H, Dubbedam A, De Wever W, et al. Predictors of Survival in Restrictive Chronic Lung Allograft Dysfunction after Lung Transplantation. J Heart Lung Transplant. 2016;35:1078–1084. doi: 10.1016/j.healun.2016.03.022 27212563

[4] Sato M, Waddell TK, Wagnetz U, Roberts HC, Hwang DM, Haroon A, et al. Restrictive allograft syndrome (RAS): a novel form of chronic lung allograft dysfunction. J Heart Lung Transplant. 2011;30:735–742. doi: 10.1016/j.healun.2011.01.712 21419659

[5] Glanville AR, Verleden GM, Todd JL, Benden C, Calabrese F, Gottlieb J, et al. Chronic lung allograft dysfunction: Definition and update of restrictive allograft syndrome-A consensus report from the Pulmonary Council of the ISHLT. J Heart Lung Transplant. 2019;38:483–492. doi: 10.1016/j.healun.2019.03.008 31027539

[6] Saito T, Horie M, Sato M, Nakajima D, Shoushtarizadeh H, Binnie M, et al. Low-dose computed tomography volumetry for subtyping chronic lung allograft dysfunction. J Heart Lung Transplant. 2016;35:59–66. doi: 10.1016/j.healun.2015.07.005 26342441

[7] Peräkylä LH, Raivio PM, Kesävuori RI, Piilonen AK, Stark CK-J, Halme MK, et al. Chronic lung allograft dysfunction subtype analysis by computed tomography volumetry. Clin transplant. 2021;36: e14507. doi: 10.1111/ctr.14507 34634164

[8] Nykänen AI, Raivio PM, Peräkylä LH, Stark CK-J, Huuskonen AS, Lemström KB, et al., Incidence and impact of chronic lung allograft dysfunction after lung transplantation–single-center 14-year experience. Scand Cardiovasc J. 2020;54:192–199. doi: 10.1080/14017431.2020.1726444 32148103

[9] Verleden GM, Glanville AR, Lease ED, Fisher AJ, Calabrese F, Corris PA, et al. Chronic lung allograft dysfunction: Definition, diagnostic criteria, and approaches to treatment-A consensus report from the Pulmonary Council of the ISHLT. J Heart Lung Transplant. 2019;38:483–492. 30962148 10.1016/j.healun.2019.03.009

[10] Levy L, Huszti E, Renaud-Picard B, Berra G, Kawashima M, Takahagi A et al. Risk assessment of chronic lung allograft dysfunction phenotypes: Validation and proposed refinement of the 2019 International Society for Heart and Lung Transplantation classification system. J Heart Lung Transplant. 2020;39:761–770. doi: 10.1016/j.healun.2020.04.012 32418864

[11] Van Herck A, Verleden SE, Sacreas A, Heigl T, Vanaudenaerde BM, Dupont LJ, et al. Validation of post-transplant chronic lung allograft dysfunction classification system. J Heart Lung Transplant. 2019;38:166–173. 30391199 10.1016/j.healun.2018.09.020

[12] Verleden SE, Von Der Thusen J, Van Herck A, Weynand B, Verbeken E, Verschakelen J, et al. Identification and characterization of chronic lung allograft dysfunction patients with mixed phenotype: a single-center study. Clin Transplant. 2020;34:e13781. doi: 10.1111/ctr.13781 31958356

[13] Verleden SE, Vos R, Vanaudenaerde BM and Verleden GM. Chronic lung allograft dysfunction phenotypes and treatment. J Thorac Disc. 2017;9:2650–2659. doi: 10.21037/jtd.2017.07.81 28932572 PMC5594185

[14] Tissot A, Danger R, Claustre J, Magnan A and Brouard S. Early Identification of Chronic Lung Allograft Dysfunction: The Need of Biomarkers. Front. Immunol. 2019;10:1681. 31379869 10.3389/fimmu.2019.01681 PMC6650588

[15] Royer P-J, Olivera-Botello G, Koutsokera A, Aubert J-D, Bernasconi E, Tissot A et al. Chronic Lung Allograft Dysfunction—A Systematic Review of Mechanisms. Transplantation 2016;100:1803–1814. doi: 10.1097/TP.0000000000001215 27257997

[16] Fuchs E, Levy L, Huszti E, Renaud-Picard B, Berra G, Kawashima M, et al. Significance of phenotype change after chronic lung allograft dysfunction onset. Transpl Intl. 2021;34:2620–2632. doi: 10.1111/tri.14157 34748217

[17] Garfield JL, Marchetti N, Gaughan JP, Steiner RM and Criner GJ. Total lung capacity by plethysmography and high-resolution computed tomography in COPD. Int J Chron Obstruct Pulmon Dis. 2012;7:119–126. doi: 10.2147/COPD.S26419 22399851 PMC3292389

[18] Horie M, Levy L, Houbois C, Salazar P, Saito T, Pakkal M, et al. Lung Density Analysis Using Quantitative Chest CT for Early Prediction of Chronic Lung Allograft Dysfunction. Transplantation. 2019;103:2645–2653. doi: 10.1097/TP.0000000000002771 31343572

[19] Byrne D, Nador RG, English JC, Yee J, Levy R, Bergeron C, et al. Chronic Lung Allograft Dysfunction: Review of CT and Pathologic Findings. Radiol Cardiothorac Imaging 2021;3: e200314. doi: 10.1148/ryct.2021200314 33778654 PMC7978021

