# Supplementary material for: Transition from BOS to RAS impairs prognosis after lung transplantation—CLAD subtype analysis by CT volumetry
Source: PLoS One. 2022 Oct 12;17(10):e0275563. doi: 10.1371/journal.pone.0275563 (PMC9555659; doi:10.1371/journal.pone.0275563)
Supplement: S1 File — A) Subclassification of CLAD at CLAD onset according to the 2019 Consensus report by ISHLT, B) subclassification distribution at CLAD onset, C) subclassification of CLAD at the end of follow-up according to the 2019 Consensus report by ISHLT, D) subclassification distribution at the end of follow-up. (DOCX) [file pone.0275563.s001.docx]

**SUPPORTING INFORMATION TO MANUSCRIPT**

| **A)** | Classification at CLAD onset, according to the ISHLT 2019 Consensus report | |  |  |  |  |  |
| --- | --- | --- | --- | --- | --- | --- | --- |
| **No** | **ISHLT classification** | **Obstruction (FEV1/FVC <0.70)** | **Restriction (FVC <80% from baseline)** | **RAS-like opacities** | **N** | **%** |  |
|  |  |  |  |  |  |  |  |
| 1 | BOS | Yes | No | No | 31 | 49 % |  |
| 2 | RAS | No | Yes | Yes | 0 | 0 % |  |
| 3 | Mixed | Yes | Yes | Yes | 0 | 0 % |  |
| 4 | Undefined | Yes | No | Yes | 4 | 6 % |  |
| 5 | Undefined | Yes | Yes | No | 13 | 21 % |  |
| 6 | Unclassified | No | No | No | 10 | 16 % |  |
| 7 | Unclassified | No | Yes | No | 3 | 5 % |  |
| 8 | Unclassified | No | NA | No | 1 | 2 % |  |
| 9 | Unclassified | Yes | NA | No | 1 | 2 % |  |

| **C)** | Classification at the end of follow-up, according to the ISHLT 2019 Consensus report | | |  |  |  |  |
| --- | --- | --- | --- | --- | --- | --- | --- |
| **No** | **ISHLT classification** | **Obstruction (FEV1/FVC <0.70)** | **Restriction (FVC <80% from baseline)** | **RAS-like opacities** | **N** | **%** |  |
|  |  |  |  |  |  |  |  |
| 1 | BOS | Yes | No | No | 16 | 25 % |  |
| 2 | RAS | No | Yes | Yes | 0 | 0 % |  |
| 3 | Mixed | Yes | Yes | Yes | 13 | 21 % |  |
| 4 | Undefined | Yes | No | Yes | 4 | 6 % |  |
| 5 | Undefined | Yes | Yes | No | 23 | 37 % |  |
| 6 | Unclassified | No | No | No | 1 | 2 % |  |
| 7 | Unclassified | No | Yes | No | 4 | 6 % |  |
| 8 | Unclassified | No | Not available | No | 1 | 2 % |  |
| 9 | Unclassified | Yes | Not available | No | 1 | 2 % |  |
